# Social network properties and self-rated health in later life: comparisons from the Korean social life, health, and aging project and the national social life, health and aging project

**DOI:** 10.1186/1471-2318-14-102

**Published:** 2014-09-14

**Authors:** Yoosik Youm, Edward O Laumann, Kenneth F Ferraro, Linda J Waite, Hyeon Chang Kim, Yeong-Ran Park, Sang Hui Chu, Won-tak Joo, Jin A Lee

**Affiliations:** 1 Department of Sociology, Yonsei University, Seoul, South Korea; 2 Department of Sociology, University of Chicago, Chicago, Illinois, USA; 3 Department of Sociology and Center on Aging and the Life Course, Purdue University, West Lafayette, Indiana, USA; 4 Department of Preventive Medicine, Yonsei University, Seoul, South Korea; 5 Division of Silver Industry, Kangnam University, Yongin, Gyeonggi, South Korea; 6 Department of Clinical Nursing Science, Yonsei University, Seoul, South Korea

**Keywords:** Social networks, Density, Centrality, Self-rated health, South Korea, USA

## Abstract

**Background:**

This paper has two objectives. Firstly, it provides an overview of the social network module, data collection procedures, and measurement of ego-centric and complete-network properties in the Korean Social Life, Health, and Aging Project (KSHAP). Secondly, it directly compares the KSHAP structure and results to the ego-centric network structure and results of the National Social Life, Health, and Aging Project (NSHAP), which conducted in-home interviews with 3,005 persons 57 to 85 years of age in the United States.

**Methods:**

The structure of the complete social network of 814 KSHAP respondents living in Township K was measured and examined at two levels of networks. Ego-centric network properties include network size, composition, volume of contact with network members, density, and bridging potential. Complete-network properties are degree centrality, closeness centrality, betweenness centrality, and brokerage role.

**Results:**

We found that KSHAP respondents with a smaller number of social network members were more likely to be older and tended to have poorer self-rated health. Compared to the NSHAP, the KSHAP respondents maintained a smaller network size with a greater network density among their members and lower bridging potential. Further analysis of the complete network properties of KSHAP respondents revealed that more brokerage roles inside the same neighborhood (*Ri*) were significantly associated with better self-rated health. Socially isolated respondents identified by network components had the worst self-rated health.

**Conclusions:**

The findings demonstrate the importance of social network analysis for the study of older adults’ health status in Korea. The study also highlights the importance of complete-network data and its ability to reveal mechanisms beyond ego-centric network data.

## Background

The National Social Life, Health, and Aging Project (hereafter, NSHAP) conducted interviews with 3,005 community-dwelling persons 57 to 85 years of age across the United States [1]. The NSHAP collected longitudinal social network data with a module that allowed respondents to provide information about their network members and the relationships among them [2]. With NSHAP data, researchers found that particular types of social networks were associated with various health-related dimensions including subjective well-being [3], depressive symptoms [3], hypertension [4], erectile dysfunction [5], health-related behaviors [6], and health-care utilization [7]. In the Korean Social Life, Health, and Aging Project (hereafter, KSHAP), we collected social network data of older Korean adults using numerous questions identical to those in the NSHAP. In addition, unlike the NSHAP, which collected data from a representative sample of American older adults, the KSHAP selected the entire population of older adults in Township K and asked them to provide the actual names of their social network members. This distinctive feature allowed us to construct a complete map of the social network of all older adults in Township K, as described below.

The KSHAP provides valuable insight into the social network structure of older Korean adults, not only by defining the social network of each respondent but also by defining the complete network of an entire village in South Korea (henceforth simply Korea). The KSHAP study adopted a multi-disciplinary approach, including social network analysis, survey interviews, physical examinations, functional assessment, and biomarker analysis to comprehensively examine the social, emotional, and physical health of older Korean adults. Detailed information about the physical examinations and biomarkers are explained in another article [8], while this study focuses on the social network properties of KSHAP. There is no previous data collected on the complete social network and biomarkers of older Korean adults and due to data limitations, no previous study has compared both the social network structure and health statuses of older adults in Korea and the United States. The unique structure of the data allows us fully to explore the differences and similarities of the social network structures and health of older adults in Korea and the United States.

Previous studies consistently demonstrated the positive effects of social networks on physical and mental health [9-11]. A person with a large social network can benefit from the positive mental effects from abundant and stable social interactions, which can be helpful to alleviate stress from negative life events, to recover from illness, and to preserve psychological and physiological health [12,13]. The patterns and compositions of social networks also determine how social and economic resources (i.e., access to medical information and services) in the community are shared and utilized by the actors [14,15]. These properties of social networks can also be crucial for the health of older adults as they experience changes in their social environments caused by retirement [16,17], bereavement due to the loss of loved ones [18,19], or diminishing physical and mental abilities [20]. While early studies focused on the dangers of reduced social engagement with age [21,22], recent studies have revealed that older adults are much more active in facing changes in their social network size when they concentrate on several high-quality social relationships [23,24], participate in more social activities [25-28], and recompose their social network structure [29-31]. Scholars are now asking specific questions about the relationship between health and the social networks of older populations, seeking to understand how older adults reform their social relations and the patterns of such relationships. To answer these questions, the KSHAP and NSHAP designed studies that provide information on the social relations of older adults in South Korea and the United States in terms of their social network size, content, composition, and structural formation.

Previously, the *size* of the social network has been considered an important factor in the health of older people. Early studies measured the amount of social activity or number of interpersonal contacts as indicators of social support [32-34] which could buffer against life stressors and preserve one’s psychological stability and health [35,36]. From this perspective, social disengagement [21,22] or isolation (i.e., perceived social isolation such as a perceived lack of social care and structurally disconnected isolation such as no close neighbors in the community) [37-39] has been treated as a crucial problem with regard to older populations. On the other hand, other studies have shown that older adults enter into new social relations to complement their network loss caused by retirement or bereavement [29] and that this activity is linked to better self-rated health and lower depressive symptoms [31]. These results show that older adults experience radical changes in their social networks. Therefore, more elaborated indices are required to capture the complex characteristics of changes in the social networks of older adults.

To explain the relationship between social network and health status, some scholars have investigated the network structure, which is closely related to the *role structure* of a community in which older adults belong. Each person occupies a certain role in the social relationship. For example, the head of a village serves in the community to maintain numerous social relations with the village’s members as well as other villages’ members. A school teacher is in a position in which she engages in frequent interactions with parents more than she does with other people in the village. This social structure provides an individual with social norms and expectations for a certain role identity [40-42]. If a person fits one’s role and acts properly based on social guidance, he or she can feel more self-esteem and a sense of mastery over life [43,44]. Previous studies have emphasized that older adults experience rapid changes in their social roles, and successful adaptation to these transitions could be linked to better health [45-47]. In this study, we focused on a specific type of social role, called a *bridging role*, which represents a network structure consisting of several actors who are not directly connected to each other and are thus expected to be more heterogeneous than a strongly integrated group of people. An older adult who performs a bridging role is advantageous in two respects. Firstly, diverse social relations provide a diverse set of social roles, which can be beneficial for older adults because it gives them additional chances to feel a sense of mastery from their role performance [48-50]. Secondly, the bridging role itself has several advantages because it provides diverse sources of information [51] and controlling power over disconnected groups [52,53]. These benefits are strongly associated with a sense of autonomy in older adults, which is a crucial factor for better health [54-56]. We used two measures of bridging roles: the *bridging potential* of the individual social networks from the KSHAP and NSHAP, and *brokerage roles* in the entire social network of the KSHAP.

Several studies have examined the effect of social network structures on health using data from Asian populations, though the results were not consistent. A positive effect of bridging roles on health was found using a representative sample from the population of Okayama, Japan [57]. In a study of 312 older Korean adults, it was found that those with diverse social networks reported better health and than those in isolated networks [58]. However, only the indirect effect of social activities and social support was found, and no relationship between social contact and mortality was observed in a national sample of older Japanese adults [59]. Also, several social network measures, such as one’s kin network or the loss of a spouse, had no effects on the health status of older Taiwanese adults, and the effects of social contacts with friends or social activities were contingent on the gender of the respondent [60]. Living alone was significantly associated with depressive symptoms and suicidal ideation in older Korean men, but not in older Korean women [61]. It can be assumed that these effects are not universal and that effects of the social network size and a bridging role on the health statuses of older adults can differ across societies and cultures. For example, in a patriarchal society in which gender role division is apparent [61,62], older men may enjoy a larger social network and occupy more bridging roles than older women. Therefore, we would like to explore the differences in the social network sizes, contents, compositions, and structures of older Korean and American adults and examine the influence of these differences on health.

To examine the relationship between the social network properties and health and to make possible an international comparison to a paper that used NSHAP data to examine the relationships between various network properties and self-rated health among older U.S. adults [2], we focus on self-rated health, the major health status measure. Self-rated health is among the most frequently adopted health status measures in epidemiological research [63]. This measure has been recommended by the World Health Organization and the European Commission for use in health monitoring [63]. Although the measurement of self-rated health needs to be adjusted by gender and culture, it is still trusted to be a valid measure of health status [64,65]. The association between self-rated health and a variety of biomarkers, such as albumin, hemoglobin, and white blood cells, have been studied and self-rated health was shown to have a graded association with all biomarkers tested; thus, we can assume that it has physiological foundations [66]. Additionally, it has long been demonstrated to predict mortality among older adults, even after controlling for physical health status and other key demographic variables [67]. In what follows, the data-collection procedures, social network module, and measurement of ego-centric and complete-network properties of the first wave of the KSHAP will be introduced.

## Data and methods

### The Korean social life, health, and aging project

The KSHAP was designed to examine the entire population (not a sample) of adults 60 years old or older and their spouses (who were not necessarily 60 years old or older) in Township K Located on Ganghwa Island, Korea. Township K is a typical rural community of Korea with ten *Ri*s, the smallest administrative unit in Korea, in one *Myeon* (Township). As of January of 2013, the total population of all ages in Township K was 1,864 (with 871 families), and with the aid of the public officers of Township K and a pilot study, a total of 860 people aged 60 or older and their spouses were identified as the KSHAP population. About 67 percent of our respondents were working, and 88 percent of them were active in farming. Ganghwa Island is 120,000 acres and is connected by two large bridges to the mainland. People usually do not notice they are on an island when they drive over the short and wide bridges. The KSHAP advertised its study to the participants by hanging banners throughout the township and by distributing study information and participation encouragement letters. The researchers also participated in several village foremen meetings, township council meetings, as well as senior citizens’ association meetings to explain the study’s purpose and solicit participation. We completed a face-to-face population survey of 60 older adults and their spouses living in Township K (814 out of 860 people) from December of 2011 to March of 2012, for a response rate of 94.65 percent. The interviews were conducted in respondents’ homes or at community centers, with an average duration of 48 minutes. The respondents were informed of the nature of the survey and informed consent was obtained prior to the survey. The study was approved by the institutional review board of Yonsei University (YUIRB-2011-012-01).

Using the population data set, a complete network of the entire community was constructed from the social network surveys. The following name generator was used to elicit discussion network members; it is identical to the one used in the NSHAP [2,68].

From time to time, most people discuss things that are important to them with others. For example, these may include good or bad things that happen to you, problems you are having, or important concerns you may have. Looking back over the last 12 months, who are the people with whom you most often discussed things that were important to you?

The social network of the respondents in this paper consists of these network members who discussed important things over the last 12 months (up to five) and a spouse if any (up to six members in total). The only difference in the social network module between the NSHAP and the KSHAP is the inclusion criteria of the spouse. The NSHAP asked the respondents to enumerate up to five discussion network members, and only if the respondent did not include his or her spouse was that person added to the additional roster. Unlike the NSHAP, the KSHAP adopted a separate roster for spouses from the beginning such that respondents with spouses listed their spouse first and then up to five discussion network members on the next roster. If the respondent had no spouse, there is no difference between the total possible number of network members in the NSHAP and the KSHAP. However, if KSHAP respondents had spouse, they could have up to six possible network members (the spouse and five discussion network members), whereas the NSHAP respondents could only have up to five possible discussion network members. Both the NSHAP and KSHAP have one extra roster for a person who was not mentioned in the previous rosters but who is very important to the respondent. However, we did not consider this roster in this analysis, concentrating on only spouses and up to five discussion network members.

Collecting network data from a sample of the population produces a number of networks from each respondent, where only the respondent and his or her own social network members are listed. This type of social network is called an *ego-centric network*, which consists of the respondent (*ego*) and their social network members (*alters*). Collecting a complete graph of the social network for all people in a specific community, however, generates all of the social relations between all of the people in a population. We refer to this type of social network as a *complete network* or a *global network*. In a complete network, we regard each person as a *node* which can be a starting or ending point of social relationships. The complete network enables us to measure three additional network characteristics of value in comparison with an ego-centric network. Firstly, we can distinguish the directions of social relations. In an ego-centric network, we can only identify which people were selected as social network members by the respondent. In the complete social network, however, we can recognize who pointed out the respondent as a member of their social network. From this information, we can differentiate *outward social relations* (with the people who were selected by the respondent) from *inward social relations* (with the people who selected the respondent). Secondly, we can also identify the network locations or positions of individuals. For example, we can identify how centrally each individual is located, or how far the one is from the other in the complete map of social relations of the community. Thirdly, with a complete network, we collect health measurements of not only the respondents, but also of the social network members of the respondents as they were all included in the survey population. Thus, the relationships between the health statuses of the respondents and those of social network members can be systematically examined.

### Social networks measures

This section discusses several crucial characteristics of network connectedness among older Korean adults. In the following, the ego-centric and complete-network measures of the KSHAP will be explained.

#### Ego-centric network measures

As described earlier, an ego-centric network comprises of two rosters: one for the spouse and the other for non-spousal social network members to whom respondents discuss important matters. The respondents were asked to identify up to five non-spousal network members. Thus, the size of the network ranges from zero to six. The network composition represents the composition ratio calculated for different types of contacts maintained by each respondent. To identify various relationship types, respondents were asked to characterize their relationship with each network member as follows: spouse, parent, child, sibling, neighbor, friend, minister/priest/other clergy, health professional, social worker, or others. Parents, children, siblings, and relatives including in-laws were recoded into a broader category of kin. The survey also asked about network members’ genders and cohabitation statuses; thus, three network composition variables are presented: the proportion of females, the proportion of kin, and the proportion of those who are cohabiting (living in the same household as the respondent).

Respondents’ emotional closeness to network members was measured by asking: “*How close do you feel your relationship is with [name]*?” Possible responses included “not very close”, “somewhat close”, “very close”, or “extremely close” (coded 1 to 4, respectively). We averaged the scores from all social network members (including spouse) to obtain a measure of average closeness to the alters. The KSHAP respondents were also asked how often they talk to each network member on an eight-point scale, ranging from “every day” to “less than once per year”. The scores were coded by assigning the approximate number of days per year that the respondent talked to the specific network member (e.g., “every day” = 365; “several times a week” = 182; “once a month” = 12; “a couple times a year” = 2) and were summed across all social network members to obtain a measure of the overall volume of contact with them. The *density* of an ego-centric network addresses the relationship among social network members by measuring the proportion of all possible pairs among social network members who know each other. We assumed two social network members did not know each other if the respondent reported they “have never spoken to each other” and counted the number of all existing social relationships between social network members in order to divide it by the number of all possible social relationships between them. *Bridging potential* refers to the likelihood that the respondent performs a role of bridging social interactions between his or her network members. We measured this with a binary variable of whether there existed any social network member in the respondent’s network who was not connected to any other network members [2,54]. Bridging potential captures the opposite characteristic of social networks in comparison with social network density. A respondent whose egocentric network is highly dense cannot occupy many bridging positions because most social network members are already connected to each other.

#### Complete-network measures

To construct a complete network of Township K based on the 814 ego-centric networks of the 814 respondents, we needed to identify the same social network members appearing in more than one ego-centric network of different respondents (i.e., duplicates). Based on the respondents’ report, the KSHAP collected detailed information to identify social network members, including the names, genders, ages, and addresses at the smallest administrative unit, the *Ri*. We assumed that two social network members were the same person if the they satisfied all of the following four criteria: 1) at least two out of three Korean characters in their names match, 2) their gender was the same, 3) their age difference was less than five years, and 4) their addresses was in the same *Ri*. After identifying duplicates, we obtained a complete network that contained 1,595 people and 2,499 social relations between them. We then excluded 583 people who were 1) not the spouses of survey respondents *and* 2) living outside Township K. A total of 1,012 nodes and 1,799 social relations were included in the analysis, and a matrix of 1,012 by 1,012 was created to calculate various network variables. Variables based on the complete network include degree centrality, closeness centrality, betweenness centrality, and brokerage roles. *Degree centrality* is the total number of social relations that a respondent has. The number of people who identify a respondent as a network member is called *in-degree centrality*, while the number of people a respondent maintains as his or her network members is called *out-degree centrality*. There exists a considerable difference between the average network size from the ego-centric networks and the average out-degree centrality in the complete network, although the two measures are identical in principle. This gap is explainable by the fact that, to build the complete network, we excluded nodes that were not spouses of the respondents *and* nodes that resided outside of Township K. *Closeness centrality* represents how closely, on average, a respondent is located to other actors in the network [69]. In a complete network, we can identify the shortest path between the respondent and another node. Closeness centrality is the reciprocal of the total length of those shortest paths. If the total length of the shortest paths becomes longer, which means the respondent is located farther away from other nodes, the value of closeness centrality becomes smaller. We used a normalized value in this paper, which is the original closeness centrality multiplied by the possible minimum value of the total length (the number of all nodes in the global network minus one, which can be observed when the respondent is connected to all other nodes in the network). The normalized form of closeness centrality varies between zero and one. *Betweenness centrality* refers to how many times the respondent appears on the shortest paths of other pairs of nodes [70]. In this analysis, we used a normalized index that ranges from zero to one, which is the original index divided by the number of all pairs of nodes except the respondent. If the respondent always appears on the shortest paths of other nodes in the global network, he or she has the highest value of betweenness centrality (one). In addition, *brokerage* measures how many times the ego occupies a bridging position through which two nodes, which are disconnected otherwise, are connected. We calculated the brokerage index based on the idea of Gould and Fernandez [71], identifying five types of brokerage roles: coordinator, itinerant, gatekeeper, representative, and liaison. Although this index captures similar characteristics of social roles to the bridging potential, it has two crucially distinctive properties. First, it considers the direction of social relations. It only concerns triadic relations which consist of “the respondent” and “the first node” which selected the respondent as its social network member and “the second node” selected by the respondent. Secondly, it also considers the group affiliation of each node. In this study, group membership was defined based on the respondent’s residential area measured at the level of *Ri*. A *gatekeeper* is someone who is identified as a network member by one outside his or her own *Ri* and who identifies another resident in the same *Ri* as that network member. A *representative* broker maintains the opposite bridging role; he or she is cited as a social network member by another resident in the same *Ri* and names another person outside the *Ri* as a network member. A *coordinator* mediates two people who are living in the same *Ri* as they are. A *gatekeeper* and a *representative* mediate and bridge different groups, performing “boundary-spanning” roles [72-75]. However, a coordinator arbitrates between relatively homogenous people in the same group. In the analysis, only the roles of coordinator, gatekeeper, and representative were examined because *itinerant* brokers (who connect two nodes in the same *Ri* but different from themselves) and *liaisons* (who are in the relations of three people from different three *Ri*s) were rare (one instance of a liaison and two itinerants).

### Self-rated health

The dependent variable is self-rated health. It was measured in five scales – poor, slightly poor, good, very good and excellent. In the analyses of the ego-centric- and complete-network variables, self-rated health was categorized into three groups – poor/slightly poor, good, and very good/excellent – and the average scores of the network variables for each category were reported. In the component analysis, we used the original self-rated-health variable measured by five scales. We calculated the average scores of self-rated health for each component. A higher value represents better self-rated health.

### Analysis

All of the statistical analyses were performed using the statistical package STATA 12. There are three tables containing the results from the statistical analyses in this paper. The first and second tables included the mean scores of the ego-centric and global network variables according to the respondents’ ages, genders, marital statuses, levels of education, self-rated health scores, and statistical significance from ANOVA tests. Overall weighted averages with sample standard deviation and skewness were also presented. The results from ego-centric networks were directly compared to the NSHAP data. The third table contained simple characteristics (e.g., mean age, gender composition, and mean social network size) of the five types of weak components in Township K. P-values from the ANOVA tests were also included in the table.

This study used cross-sectional data from older adults in Township K; therefore, we were not able to determine causal directions but could only identify some correlations between social network characteristics and health. Previous studies stated that social network position may be influenced by the health statuses of older adults [7,54], suggesting that the causal relationship between social network structure and health could be reversed or bi-directional. We interpreted both possibilities of causal directions for the analyses and provided explanations of several special cases in which the causal direction was relatively clear.

## Results

### Ego-centric network characteristics of Township K

Table 1 reveals that, on average, older people and their spouses in Township K maintained 3.07 network members. Older adults with a larger social network were more likely to be younger, male, living with their spouse, highly educated and have better self-rated health. The proportion of females within a network was positively associated with age and negatively related to education and self-reported health status. Also, females or respondents who were not living with a spouse (i.e., unmarried, separated, divorced, or widowed) had social networks with a higher proportion of females. The proportion of kin showed no significant differences across all characteristics except marital status. The proportion of cohabiting social network members was higher when the respondents were male, living with their spouse, or highly educated. People who felt closer to their social network members reported that they were living with their spouse and had better self-rated health. A higher volume of contact with social network members was observed from people who were male, living with their spouse, only educated in elementary school or a *seodang* (traditional village-based elementary school in Korea), or had better self-rated health. The average density among network members of older people in Township K was 0.98, indicating that they maintained tightly connected, dense networks. Males showed denser networks than females, although the discrepancy was not great, at 0.99 to 0.97. On average, older people in Township K had very low bridging potential (0.01), which is natural given the extremely high density, as discussed above. Only one out of 100 people had a network member who was not connected to any other members in his or her network. The respondents who were males or living with their spouses had more bridging potential than other groups.

**Table 1:** **Network measures by selected variables in the KSHAP and NSHAP: ego-centric network**^**a**^

			**Network Size (spouse + alters)**		**Proportion of female**		**Proportion of kin**		**Proportion of cohabitation**		**Average closeness to alters**		**Overall volume of contact (per year)**		**Network density**		**Bridging potential**	
	**KSHAP (N, proportion)**	**NSHAP (N, proportion)**	**KSHAP**	**NSHAP**	**KSHAP**	**NSHAP**	**KSHAP**	**NSHAP**	**KSHAP**	**NSHAP**	**KSHAP**	**NSHAP**	**KSHAP**	**NSHAP**	**KSHAP**	**NSHAP**	**KSHAP**	**NSHAP**
Age	≤64		3.25	3.71	0.48	0.61	0.55	0.68	0.33	0.30	3.31	3.21	717.28	780.34	0.99	0.86	0.00	0.06
	(151, 0.19)	(1020, 0.34)																
	65-74		3.17	3.65	0.53	0.62	0.55	0.68	0.30	0.28	3.28	3.15	717.26	720.25	0.98	0.84	0.01	0.08
	(367, 0.45)	(1092, 0.36)																
	≥75		2.85	3.57	0.60	0.64	0.54	0.70	0.30	0.22	3.26	3.11	668.97	690.48	0.98	0.84	0.02	0.09
	(296, 0.36)	(893, 0.30)																
	p-value		0.00	0.27	0.00	0.20	0.84	0.26	0.37	0.00	0.75	0.00	0.23	0.00	0.59	0.07	0.35	0.23
Gender	Male		3.28	3.41	0.42	0.57	0.55	0.70	0.34	0.35	3.25	3.14	734.53	675.00	0.99	0.87	0.00	0.06
	(342, 0.42)	(1455, 0.48)																
	Female		2.92	3.88	0.64	0.67	0.55	0.67	0.29	0.20	3.30	3.19	674.47	797.33	0.97	0.84	0.02	0.09
	(472, 0.58)	(1550, 0.52)																
	p-value		0.00	0.00	0.00	0.00	0.95	0.17	0.00	0.00	0.29	0.02	0.03	0.00	0.02	0.00	0.01	0.01
Marital status	Living with spouse		3.33	3.75	0.48	0.59	0.60	0.74	0.37	0.36	3.32	3.20	760.91	780.08	0.98	0.88	0.01	0.03
	(612, 0.76)	(1801, 0.60)																
	Separated/divorced/widowed		2.31	3.46	0.76	0.69	0.39	0.57	0.12	0.09	3.16	3.10	523.38	653.91	0.96	0.79	0.03	0.16
	(196, 0.24)	(1204, 0.40)																
	p-value		0.00	0.00	0.00	0.00	0.00	0.00	0.00	0.00	0.00	0.00	0.00	0.00	0.08	0.00	0.00	0.00
Education	None	<High school	2.77	3.19	0.63	0.65	0.55	0.74	0.28	0.30	3.29	3.17	647.06	722.69	0.99	0.88	0.01	0.07
	(242, 0.30)	(699, 0.23)																
	Elementary school/Seodang^b^	High school or equivalent	3.11	3.55	0.53	0.63	0.53	0.73	0.31	0.27	3.25	3.19	746.87	750.14	0.98	0.88	0.01	0.07
	(334, 0.41)	(793, 0.26)																
	≥Middle school	Some college	3.33	3.79	0.48	0.62	0.57	0.66	0.34	0.26	3.33	3.16	689.66	756.31	0.97	0.84	0.01	0.08
	(230, 0.29)	(856, 0.28)																
		≥Bachelor’s		3.96		0.59		0.64		0.27		3.14		713.92		0.81		0.08
		(657, 0.22)																
	p-value		0.00	0.00	0.00	0.02	0.51	0.00	0.01	0.25	0.34	0.42	0.01	0.18	0.23	0.00	1.00	0.97
Self-rated health	Poor/Somewhat Poor		2.85	3.48	0.57	0.63	0.57	0.70	0.30	0.26	3.20	3.10	646.11	736.80	0.98	0.85	0.01	0.08
	(353, 0.43)	(806, 0.27)																
	Good		3.21	3.54	0.52	0.62	0.53	0.70	0.31	0.29	3.32	3.17	739.63	732.18	0.97	0.86	0.01	0.08
	(399, 0.49)	(906, 0.30)																
	Very good/excellent		3.42	3.83	0.53	0.62	0.55	0.67	0.31	0.27	3.47	3.20	747.89	742.33	0.99	0.85	0.00	0.07
	(62, 0.08)	(1,281, 0.43)																
	p-value		0.00	0.00	0.06	0.95	0.32	0.06	0.71	0.07	0.00	0.02	0.00	0.81	0.65	0.76	0.70	0.49
Overall weighted mean			3.07	3.65	0.55	0.62	0.55	0.69	0.31	0.27	3.28	3.16	699.70	738.04	0.98	0.85	0.01	0.08
SD			1.23	1.47	0.29	0.27	0.36	0.31	0.24	0.27	0.66	0.51	386.43	354.39	0.12	0.23	0.10	0.26
Skewness			0.43	−0.42	0.04	−0.28	0.08	−0.61	1.11	1.44	−0.57	−0.20	1.04	0.57	−6.81	−1.60	9.54	3.22

^a^The early publication of NSHAP [2] only considered the roster of discussion network members (roster A). In this analysis, however, the roster of spouse (roster B) was also considered.

^b^Seodang is a traditional village-based elementary school in Korea.

Focusing on the relationship between social network characteristics and self-rated health, older adults who interacted more frequently with and felt closer to a larger number of social network members reported better self-rated health. Stronger, more intimate, more communicative social relationships can generate more social support, which would be beneficial for maintaining good health and recovering from illness. Conversely, we can consider the reverse effect – healthier people could be inclined to participate in more social activities and interact with more people. However, we assume that social network density and bridging potential are not likely to be influenced by the health statuses of the respondents given that the social relations among one’s network members are more difficult to manage or change than managing the dyadic relationship between the respondent himself and his social network members. Especially in such a densely integrated social community as Township K, a person has difficulty in occupying bridging positions because there are few disconnected social groups to bridge. In this analysis, we could not estimate the impact of the bridging potential on self-rated health because there was little variation among the population. Future studies with longitudinal data will complement these types of limitations by examining the effects of changes in social network sizes and structural formations on health statuses.

The results from the NSHAP are presented in Table 1 to compare the network properties of older American adults [2], and they reveal several interesting dissimilarities between older people in Township K of South Korea and a representative sample of older adults in the U.S. For those with a spouse, the KSHAP mandatorily includes their spouse as a member of their network, unlike the NSHAP, where the inclusion of a spouse as a network member was not mandatory. Therefore, it is assumed that the KSHAP respondents would have a larger network size on averages especially when 70 percent of the KSHAP respondents are living with their spouse, compared to the NSHAP, where 60 percent live with their spouse. However, when compared to older U.S. adults, older adults in Township K maintained smaller (3.07 vs. 3.65) but denser (0.98 vs. 0.85) social networks. As a result, KSHAP respondents had a lower volume of contact with network members (699.70 vs. 738.04) and a smaller bridging potential (0.01 vs. 0.08). In addition, older men in the NSHAP had smaller networks (3.41 vs. 3.88) and lower volumes of contact (675.00 vs. 797.33) when compared to older women in the NSHAP. In sharp contrast, older Korean men enjoyed larger networks (3.28 vs. 2.92) and more frequent contact with network members (734.53 vs. 674.47) when compared to the older women in the KSHAP. In both countries, however, people with larger social networks reported better self-rated health.

### Complete-network characteristics of Township K

In Table 2, both in-degree and out-degree centrality were higher among people between the ages of 65 and 74 and who were living with their spouse. In-degree centrality was positively correlated with self-rated health, which indicates that older adults who were selected as social network members by other residents in Township K were likely to have better self-rated health than those who were not selected. More inward social relations could be linked to more social concern and aid from neighbors, which could be beneficial for older adults as it may help them to maintain better health. Inversely, it may be that people with better health are more capable of consulting on important matters and managing social relations and therefore are more likely to be cited as a social network member by neighbors. Closeness centrality was higher when the respondents were aged 75 or above, living with a spouse, and had an only elementary school education or were educated in a *seodang*; however, this was not significantly correlated with self-rated health. Betweenness centrality showed no significant differences among all categories except marital status. We found a consistent association between a large social network centrality and cohabitation with a spouse. As noted above, the survey design of the KSHAP enabled respondents to have one more roster for social network members, which could have affected the results of social network centrality. Although we could not effectively separate the impact of the survey design from marital status in this analysis, we could expect some benefits of marriage with regard to enjoying active social relationships with other people.

**Table 2:** Network measures by selected variables in the KSHAP: Complete network

		**In-degree centrality**	**Out-degree centrality**	**All-closeness centrality**	**Betweenness centrality**	**Brokerage: coordinator**	**Brokerage: representative**	**Brokerage: gatekeeper**
Age	≤64	1.95	2.16	0.0600	0.0022	2.83	0.18	0.25
	65-74	2.09	2.26	0.0669	0.0022	3.34	0.12	0.09
	≥75	1.71	2.18	0.0678	0.0016	2.36	0.29	0.13
	p-value	0.01	0.65	0.07	0.48	0.01	0.18	0.09
Gender	Male	2.09	2.40	0.0683	0.0023	3.33	0.35	0.25
	Female	1.81	2.07	0.0642	0.0018	2.57	0.08	0.05
	p-value	0.01	0.00	0.10	0.27	0.01	0.00	0.00
Marital status	Living with spouse	2.10	2.36	0.0678	0.0023	3.31	0.23	0.17
	Separated/divorced/widowed	1.39	1.76	0.0604	0.0009	1.65	0.07	0.01
	p-value	0.00	0.00	0.01	0.01	0.00	0.10	0.01
Education	None	1.78	2.03	0.0679	0.0021	2.29	0.19	0.05
	Elementary school/Seodang	2.04	2.38	0.0675	0.0023	3.45	0.23	0.18
	≥Middle school	1.91	2.14	0.0613	0.0015	2.73	0.11	0.12
	p-value	0.16	0.00	0.07	0.35	0.00	0.50	0.10
Self-rated health	Poor/Somewhat Poor	1.74	2.10	0.0632	0.0016	2.50	0.11	0.05
	Good	2.01	2.34	0.0684	0.0024	3.19	0.28	0.19
	Very good/excellent	2.44	2.00	0.0654	0.0014	3.21	0.10	0.19
	p-value	0.00	0.01	0.13	0.19	0.06	0.13	0.02
Overall weighted mean		1.92	2.21	0.0659	0.0020	2.89	0.19	0.13
SD		1.60	1.29	0.0358	0.0065	4.10	1.18	0.73
Skewness		1.48	0.73	−0.93	5.58	2.56	10.01	7.14

With regard to brokerage roles, coordinators — people who occupy bridging positions inside the same *Ri* — were more likely to be aged 65 to 74, male, with an elementary school education, and with better self-rated health. Two other types of brokers, the positions of gatekeepers and representatives — who bridge insiders and outsiders for their Ri — were more likely to be occupied by older men. Older adults who were in better self-rated health typically performed these roles more often; however, more gatekeeper positions were occupied by people with ‘good’ self-rated health than with ‘poor’ or ‘very good’ self-rated health. It is noteworthy that brokerage roles in Township K were strongly correlated with self-related health when the brokers were coordinating people in the same *Ri* compared to when they were mediating and bridging people from different *Ri*s. Previous studies noted that the advantages of mediating and bridging different groups were highly contingent on environmental factors [74,76,77]. Thus, it is feasible that bridging and mediating people from other *Ri*s may involve mental and physical demands that older people may find stressful and challenging such that no health-related advantages of bridging roles can be found. Future studies investigating this issue and focusing on the social meaning of living in different *Ri*s in Township K will be helpful to understand the effects of each brokerage type on health.

**Figure 1:**
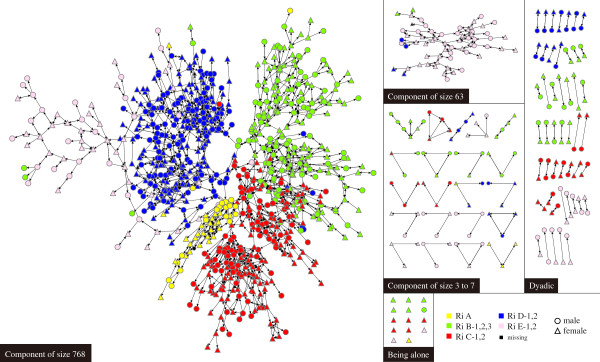
Attributes of the complete social network of older adults in Township K.

For a better understanding of group partitioning in Township K, we performed a component analysis, which is possible with complete-network data. A component is a group that is connected within and separated between groups [78]. If there is a direction in a network, two types of components are possible: weak and strong. A strong component is a group in which every person is reachable by every other person following the directions of the discussion network. A weak component is a group in which every person is reachable by every other person but not necessarily following the directions of the network. We used weak components because there were too many strong components (537) in the village; moreover, we believe strong components are too restrictive when used to measure the social dynamics of the village. Figure 1 illustrates and Table 3 summarizes these 85 weak components of Township K. In order to describe the characteristics of the weak components, we categorized them into five types - the largest component of 765 nodes, the second largest component of 63 nodes, the components of 3 to 7 nodes, dyadic, and lone nodes with no social network member. Because using all 10 *Ri*s creates a cluttered appearance, we used five *Ri*s instead. These five categories were not chosen arbitrarily but were based on the official formation of Township K, when there were only five *Ri*s; the government later ramified these five into 10 by dividing most into two or three *Ri*s. Thus, for example, *Ri* B-1, *Ri*-B-2, and *Ri* B-3 are contiguous and quite homogeneous.

**Table 3:** **Selected characteristics of each component in the KSHAP**^**ab**^

**Component**	**Component size**	**No. of nodes**	**No. of respondents**	**Age**	**% female**	**No. of chronic disease**	**Years R lived in village**	**Network size**	**% married**	**Self-rated health**^**c**^	**% of nodes in *Ri *** **A**	**% of nodes in *Ri *** **B**	**% of nodes in *Ri *** **C**	**% of nodes in *Ri *** **D**	**% of nodes in *Ri *** **E**
Size 768	768	768	624	72.465	0.575	2.216	52.144	3.151	0.771	2.590	0.060	0.266	0.267	0.323	0.081
Size 63	63	63	49	70.531	0.571	1.449	34.449	2.857	0.816	2.245	0.000	0.032	0.000	0.063	0.889
Others	3 to 7	71	44	69.750	0.636	1.818	33.227	2.455	0.651	2.477	0.042	0.324	0.155	0.169	0.310
Dyadic only	2	96	83	71.337	0.530	1.88	34.618	3.084	0.805	2.554	0.000	0.313	0.250	0.229	0.208
Being Alone	1	14	14	74.357	0.929	2.286	45.786	2.071	0.000	2.429	0.071	0.429	0.357	0.000	0.143
	Overall Mean			72.119	0.580	2.072	48.160	3.070	0.757	2.557	0.049	0.262	0.242	0.283	0.160
	p-value			0.078	0.076	0.032	<.001	<.001	<.001	0.051	0.036	<.001	<.001	<.001	<.001

^a^All descriptive statistics, except % of nodes in each component, were calculated based on information of the survey respondents.

^b^4 nodes who were spouses of the survey respondents but did not participate in the survey were not the residents of Township K.

^c^Self-rated health was measured by 5 scales, and the scores were averaged by each component (higher value represents better self-rated health).

As shown in Table 3, the largest component has 768 members and includes residents from all five *Ri*s. In sharp contrast, the second largest component has 63 members, about 90% of whom belong to one *Ri*, *Ri* E. Thus, although the component with 63 people was the second largest component of Township K, it mainly consisted of residents in specific *Ri*s: *Ri* E-1 and E-2. In that sense, it was socially segregated from the rest of Township K. *Ri* E is also geographically isolated. It is located on the west end of the Township K and thus faces the sea on the left. An army base is located north of *Ri* E, and people have to pass a military checkpoint in order to enter the *Ri*. Interestingly, the residents in this component reported the lowest self-rated health; their average score was 2.25, even lower than that of residents who had no spouse and no social network members in Township K. The majority of the residents (65%) reported “poor” or “somewhat poor” self-rated health, and no one reported “very good” or “excellent” self-rated health. In this case, we can find causal direction from social network to individual health, considering that it is difficult for one’s health status to change the structural connectedness of the entire community to which one belongs. The structural segregation of the people in the second largest component that could be related to a lack of social support and restricted diffusion of information, which could be highly associated with the low average health status score in the isolated component.

## Discussions

By studying the social network characteristics of an entire township in rural Korea, the KSHAP revealed a series of new findings based on two-level network data from ego-centric and global-level (complete) networks. Based on the ego-centric network characteristics of the residents, it produced several noteworthy results. Firstly, both older Korean and American adults had better self-rated health when they had larger networks, as was found in previous American studies [2,79]. Secondly, although there was no age difference in the number of network members among American adults, there was a notable difference in network size by age among older Korean adults. Unlike older American adults who maintained a certain social network size as they became older, older Korean adults experienced a decrease in their network size as they grew older. Third, while older American women reported a larger network size than older American men, older Korean women reported a smaller network size than older Korean men. This may reflect the strong patriarchal norms in rural Korean villages, where people are confined to their socially constructed roles: men as breadwinners and women as family care providers [61,62]. Unlike the United States, where older women can find many opportunities to sustain a large number of social network members, Korean women in rural villages may still be constrained by household chores and farming, even when they become older adults. These differences reveal that aging processes are not universal across different societies and cultures and that the developing pathways of aging may be specific to the gender and societal milieu (including societal norms) of the older adults. Fourthly, on average, older Korean adults maintained smaller but denser networks than those of older American adults. For rural older Korean adults, social network members were mainly limited to their relatives living close by, but American older adults maintained more relationships with people other than their relatives. We cannot generalize our findings to differences between USA and Korea, as the KSHAP was limited to a traditional rural village. The social network characteristics of urban older adults in Korea would be worth examining in future studies.

According to the results from complete-network analysis of the KSHAP, older adults selected as social network members by more neighbors in Township K were likely to have better self-rated health. We also examined different types of brokerage positions and found that people who were mediating between two other people within the same *Ri* (coordinator) reported better self-rated health, whereas bridging roles between people across different *Ri*s (gatekeeper and representative) were not associated with self-rated health. In general, social network analysis assumes that the brokerage position secures the advantage of being exposed to different types of social norms, information, and beliefs. We believe, however, in Township K, this advantage is limited to the same neighborhoods (*Ri*s). In other words, brokerage positions that are still well embedded in one neighborhood (*Ri*) are more helpful than those that span different neighborhoods (*Ri*s).

In order to explain these mixed effects of brokerage positions, a more detailed study about the social meaning(s) of administrative districts in Township K is required. We also conducted a component analysis based on the complete-level network data, yielding entirely new types of findings. The residents of a socially isolated component tended to report the worst self-rated health. This result is interesting considering that this component consisted of 63 people, the second largest component. In addition, the mean size of social network of the people in the component size of 63 (2.86) was third highest out of the five types of components, as shown in Table 3. This signifies that even when a person maintains a social network of an average size and belongs to a relatively large group (component), they may still report poor self-rated health if the group to which they belong is relatively segregated from the rest of the village.

## Conclusions

The results from the component analysis offer three contributions to the scholarship on social network and health. Firstly, this component-level segregation is not equal to the concepts of ‘loneliness’ or ‘isolation’ examined in previous studies. The component-level segregation in this study is different from perceived loneliness [38,80] or isolation as measured by the absence of contacts [81,82]. Even if a respondent enjoyed many social network members and thus did not feel lonely, when the group to which they belonged was segregated from the rest of the village, their self-rated health status could be worse off than people in the larger village network. Only global-level network data can identify this type of social segregation. Secondly, the segregation of components enables researchers to reconsider the neighborhood effects on health statuses. Prior studies considered several characteristics of communities, such as social trust [83,84], socio-economic status [85-87], racial segregation [88], and perceived neighborhood environment [87,89] as predictors of self-rated health. In those studies, researchers defined a boundary of a neighborhood based on administrative districts (e.g., census tracts). A component analysis, however, gives us a concrete picture of group segregation based on social relations, which could be used to redefine what the neighborhood is. Furthermore, this map of segregation shows how *Ri*s are socially linked and separated, which could be helpful for capturing the social meaning(s) of boundary-spanning roles in Township K. Future studies will be able to examine the systematic effects of social relations using KSHAP data – from individuals to components – and from social relations within a *Ri* to the bridging roles between *Ri*s.

Our causal conclusions are limited because we only examined the first-wave data of the KSHAP. With cross-sectional data, we are very limited in our ability to establish a causal direction between self-rated health and network characteristics. While network characteristics have shown systematic effects on diverse aspects of health status in numerous studies, it is also possible that health status has an effect on network figures. For example, older adults who have serious health problems and thus are not ambulatory would be expected to have difficulty maintaining rich social relationships. In the component analysis, however, a clear causal relationship from social network to individual health was found, revealing that the structural segregation of the people in the second largest component had the lowest self-rated health.

Since the KSHAP study only surveyed a rural Korean township, the study findings and contributions should be carefully evaluated. According to another study which collected ego-centric network data of older Korean adults in an urban area [90], there was no substantial difference between the KSHAP sample and urban Korean older adults in terms of their social network characteristics. With these considerations in mind, a desirable next step would be to observe the association between health and social networks among older adults in urban settings and to examine longitudinal changes of the correlations in both urban and rural data. Second-wave data of the KSHAP has been collected and we are trying to expand recruitment to neighboring communities as well as urban areas. The longitudinal analysis of social network and health of older adults will be conducted in the future studies.

### Nomenclature

Ego: The person who identified other people with whom he or she has social relations. All survey respondents become egos.

Alter: A person who was identified by an ego.

Node: All people appearing in the network, including egos and alters. It is visualized as a dot (o) on the network graph.

Tie: The social relationship between nodes. It is visualized as a straight directional line (→) on the network graph.

Ego-centric network: Local network of one ego and his or her alters.

Complete network: Complete network of all nodes. It can be created only from the data of the entire population.

Component: A group of nodes connected by at least one tie.

## Competing interests

The authors declare that they have no competing interests.

## Authors’ contributions

YY planned with the study, supervised the data collection and data analysis, and wrote the paper. EOL helped with the study plan and revised the manuscript. KFF helped with the study plan and revised the manuscript. LJW helped with the study plan and contributed to revising the paper. HCK helped with the study plan and revised the manuscript. YP helped with the study plan and contributed to revising the paper. SHC helped with the study plan and contributed to revising the paper. WJ performed statistical analyses and contributed to revising the paper. JAL performed statistical analyses and contributed to revising the paper. All authors read and approved the final manuscript.

## Pre-publication history

The pre-publication history for this paper can be accessed here:

http://www.biomedcentral.com/1471-2318/14/102/prepub
